# Controlling the Size and Pattern Pitch of Ni(OH)_2_ Nanoclusters Using Dip-Pen Nanolithography to Improve Water Oxidation

**DOI:** 10.3390/molecules25122937

**Published:** 2020-06-26

**Authors:** Zorik Shamish, Moshe Zohar, Dror Shamir, Ariela Burg

**Affiliations:** 1 Department of Chemical Engineering, Shamoon College of Engineering, P.O. Box 950, Beer-Sheva 8410802, Israel; zoriksh@gmail.com; 2 Nuclear Research Center, Negev, P.O. Box 9001, Beer-Sheva 8419001, Israel; drorshamir@gmail.com; 3 Department of Electrical and Electronics Engineering, Shamoon College of Engineering, P.O. Box 950, Beer-Sheva 8410802, Israel; moshezo@ac.sce.ac.il

**Keywords:** alternative energy, dip-pen nanolithography, meta-chemical surface, Ni(OH)_2_, water-splitting process

## Abstract

We use dip-pen nanolithography to accurately pattern Ni(OH)_2_ nanoclusters on a metachemical surface with an exceptionally large surface area. The distance between the nanoclusters can be manipulated to control the oxygen-evolution reaction current and overpotential, thereby improving the efficiency of the water-splitting process while using minute amounts of the catalyst.

## 1. Introduction

The production of hydrogen in a water-splitting process (WSP) is one of the most promising sources of alternative, environmentally friendly energy, Reaction (1) below [1,2,3,4,5,6]. An important step in this process is the oxygen-evolution reaction (OER) [5,7,8,9], which is typically conducted electrocatalytically [10,11,12], photocatalytically [10,13], or electro-photocatalytically [10].

(1) $$2H_{2}O\;\overset{}{\to }O_{2}+2H_{2}$$

The formation of O${}_{2}$ from water requires a significant molecular rearrangement and is a challenging process, both kinetically and thermodynamically [14,15]. Thus, intense efforts are made to increase its efficiency, so as to increase the efficiency of the hydrogen-evolution reaction (HER) in the generation of alternative, green energy. Several aspects should be considered when attempting to improve the efficiency of the oxidation process. First, heterogeneous catalysis is favored over homogenous catalysis [11,13,16,17,18,19], as the former allows the recycling of the catalyst and renders the issue of catalyst solubility irrelevant [16,20]. Second, the over-potential of the OER—which is usually high—needs to be as low as possible to reduce energy consumption [14,21,22,23,24,25]. Third, the catalyst should be inert, such that ligands do not oxidize at a potential that is lower than that of the OER [16]. Fourth, the catalytic process should be selective [24], which requires that the catalyst clusters are uniform and the distance between them is tightly controlled [21,26], as the surface carrying the clusters often plays a role in the OER [27,28]. Finally, because heterogeneous catalysis occurs on the surface of the catalyst, this surface area needs to be large, i.e., nano-sized catalysts are preferred over larger ones. To date, most processes that enable the formation of such nano-sized catalysts are expensive, involve a complicated production process (numerous steps are required to reach the nano-scale size), and do not enable full control over the size distribution of the nanoclusters or the distance between them (the pattern pitch), resulting in large variability that reduces the efficiency of the WSP [29,30].

We propose a novel, simple, and cost-efficient method for the accurate patterning of uniform, nano-sized catalyst clusters, using minute amounts of the catalyst [31]. We employ the NLP2000 (NanoInk, Inc.) dip-pen nanolithography (DPN) platform [31], which enables tight control over the size of the clusters and the pattern pitch dimension, as well as over the concentration of the catalyst clusters. Notably, the same method can be used to pattern any active material [31], and is, therefore, relevant to numerous applications—including the OER. Here, as a model catalyst for the OER, we chose to use Ni(OH)_2_—a cheap and widely used catalyst in this process [21,26,32,33,34,35,36,37]—although the proposed method can be used to pattern a wide range of catalysts. To form the catalytic surface, we patterned the catalyst so as to produce a meta-chemical surface (MCS), which is somewhat analogous to the widely used meta-surface [38]. Specifically, while any periodic two-dimensional surface can be defined as a meta-surface if its thickness and periodicity are small as compared with the wavelengths in its surroundings, an MSC is defined as either a periodically or a randomly patterned meta-surface in which at least one of the patterned elements is in the nanoscale range [39]. While the common meta-surfaces enable a wide range of unique optical behaviors that are not commonly found in nature, MCSs have unique chemical properties that do not occur in the bulk form of the material. In addition, while most meta-surfaces are made of semiconductor materials and metals [40,41], MCSs can be manufactured from liquid inks, including polymers, proteins, and, as shown in this work, salt solutions. To pattern the catalyst on the surface, we used the accurate and affordable DPN technique—here, employing the NLP2000 DPN tool [31].

## 2. Results and Discussion

Our study comprised two consecutive steps. First, even though DPN was already being used for patterning of metal oxides [42], the NLP2000 tool has not been previously used for patterning in aqueous solutions. For that reason, we determined the optimal patterning conditions and characterized the patterned surfaces using atomic force microscopy (AFM; Easy Scan 2 Flex, NanoSurf, Liestal, Switzerland), scanning electron microscopy (SEM FEI, Thermo Fisher Scientific, various 400L, Hillsboro, OR, USA), energy-dispersive X-ray spectroscopy (EDS X-Max${}^{N}$ 80, Oxford Instruments, Oxford, UK), and X-ray diffraction (XRD; Panalytical B.V., Almelo, The Netherlands).

Based on the optimal conditions elucidated, we then patterned Ni(OH)_2_ clusters on an indium tin oxide surface (ITO, 30–60 Ω/sq) to produce two types of MCS as electrodes for heterogeneous OER: MCS${}_{\mathrm{Ni}}$ Type 1 and MCS${}_{\mathrm{Ni}}$ Type 2, which differ in their pattern pitch dimensions, Table 1.

To pattern the clusters, we used an M-type probe (NanoInk, Inc.), which comprises 12 cantilevers whose tips are spaced 66 μm apart (the tips were cleaned in oxygen plasma before use). We studied the activity of each MCS${}_{\mathrm{Ni}}$ electrodes in the OER by linear sweep voltammetry (LSV), using the pocketSTAT instrument (Ivium Technologies, The Netherlands). The electrochemistry cell included Pt as the counter electrode, Ag/AgCl as a reference electrode, and the MSC as the working electrode. All the measurements were done in the presence of Ar.

**Figure 1:**
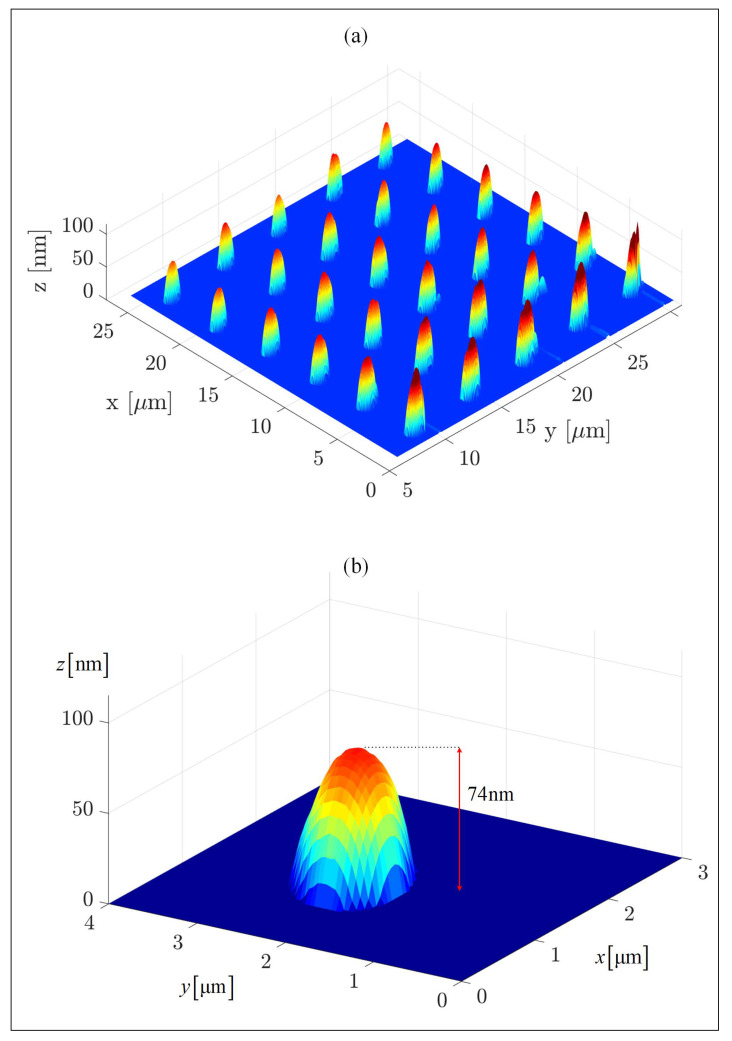
The patterned MSC${}_{\mathrm{Ni}}$ surface. (**a**) A MATLAB analysis of an AFM scan of the MCS${}_{\mathrm{Ni}}$ Type 2 electrode (patterning conditions were 0.3 ppm of Ni(OH)_2_ ink, 80% humidity, 20 ${}^{\circ }$C). The patterning was performed with an M-type cantilever. (**b**) A single cluster patterned on the electrode. The measured RMS roughness parameter of the pre-pattern indium tin oxide (ITO) surface is $S_{q}=2.572$ nm.

**Figure 2:**
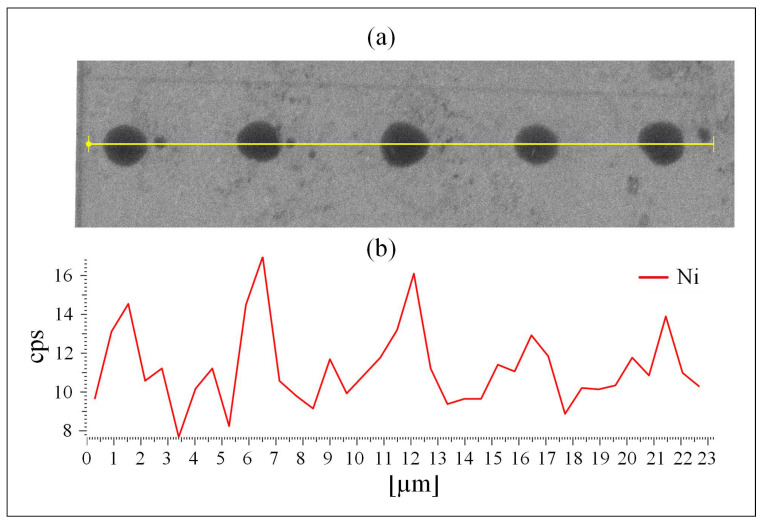
The patterned MCS${}_{\mathrm{Ni}}$ Type 2 surface. (**a**) SEM image and (**b**) EDS mapping of the MSC${}_{\mathrm{Ni}}$ nano clusters of Ni(OH)_2_ on the ITO surface.

MATLAB-analyzed AFM scans of the patterned MCS${}_{\mathrm{Ni}}$ Type 2 electrodes, Figure 1a, as well as SEM scans, Figure 2a and EDS measurements, Figure 2b, of this electrode, demonstrate the uniform patterning of the nano-scale Ni(OH)_2_ clusters on the MCS${}_{\mathrm{Ni}}$ and indicate that the clusters comprise Ni. The diameter and height range of the clusters, as measured with AFM, are 1–6 μm and 25–360 nm, respectively, and the pattern pitch dimensions are 5 μm and 4.4 μm for the *x*- and *y*-axis, respectively.

Figure 1b shows a single patterned dot (namely, a single Ni(OH)_2_ cluster) that was chosen from MCS${}_{\mathrm{Ni}}$ Type 2. The base of this cluster is elliptical due to the pyramid shape of the tip, the patterning mechanism, and the cohesive–adhesive forces: while the cohesive forces between the Ni(OH)_2_ units are stronger than the adhesive forces between the ink and the ITO surface, the cohesive forces between the Ni(OH)_2_ units on the top edge of the cluster are weaker than the adhesive forces between the ink and the probe, resulting in elliptical (rather than circular) clusters. The average length of the elliptical bases of the clusters are a=0.573 μm and b=0.523 μm for the semi-major and semi-minor axes, respectively, and their average height is 74 nm. The average surface area and the approximate average volume of the clusters are S=0.961 (μm${)}^{2}$ and V=0.043 (μm${)}^{3}$, respectively, resulting in a surface area to volume ratio of 22.57 (μm${)}^{-1}$. Such a higher ratio can explain the improved efficiency of the WSP (see below), despite the use of a minute amount of Ni(OH)_2_.

XRD measurements conducted after the LSV measurements indicate the formation of nickel oxide on the surface. Together with the results of the LSV, this finding is in line with the mechanism suggested in the literature [27,43] for the electrocatalytic WSP and the formation of NiOOH. Reactions (2) and (3), below, are suggested for the HER and the nickel oxidation [27,43], respectively:

(2) $$H_{2}O+e^{-}\overset{}{\to }\frac{1}{2}H_{2}+{\mathrm{OH}}^{-}$$

(3) $$\mathrm{Ni}{\left(\mathrm{OH}\right)}_{2}+{\mathrm{OH}}^{-}\overset{}{\to }\mathrm{NiOOH}+H_{2}O+e^{-}\;\;\left(\mathrm{occurs}\;\mathrm{on}\;\mathrm{the}\;{\mathrm{MCS}}_{\mathrm{Ni}}\right)$$

Reactions (4) and (5), below, are suggested for the nickel reduction and OER [27,28,29], respectively:

(4) $$\mathrm{NiOOH}+H_{2}O+e^{-}\overset{}{\to }\mathrm{Ni}{\left(\mathrm{OH}\right)}_{2}+{\mathrm{OH}}^{-}$$

(5) $$2{\mathrm{OH}}^{-}\overset{}{\to }\frac{1}{2}O_{2}+H_{2}O+2e^{-}$$

**Figure 3:**
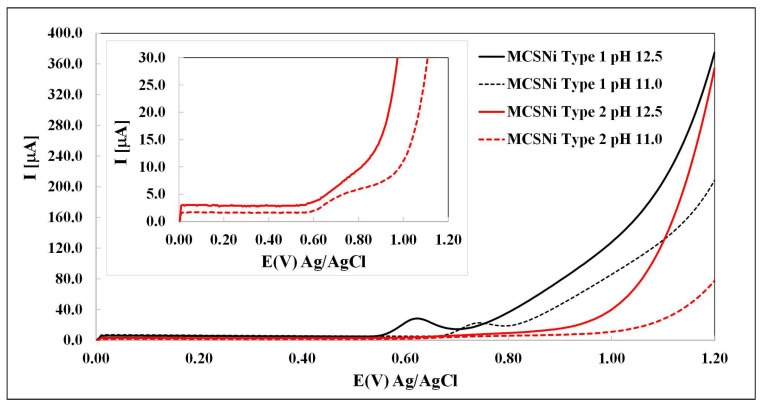
Linear sweep voltammetry of MCS${}_{\mathrm{Ni}}$ as a working electrode in 0.20 M NaClO_4_, 1000 mV/s. The two types of electrode are indicated by line color (black: Type 1, red: Type 2) and the two pH values are indicated by line pattern (solid: pH 12.5, dashed: pH 11.0, Ar atmosphere).

The activity of each of the two types of MCS${}_{\mathrm{Ni}}$ was studied by LSV in 0.20 M NaClO_4_ solutions under various pH conditions (from pH 8 to pH 12.5, Ar atmosphere), so as to be able to compare our findings with those reported previously [27,43]. As expected [22,27,40], we found that the oxidation process occurs at pH > 10, and that the oxidation current and potential depend both on the pH and on the pattern pitch dimension (Figure 3). The waves between ∼0.6–0.7 V (depending on the conditions) are attributed to the oxidation of Ni(OH)_2_ through Reaction (3). Our findings indicate that (i) as expected [44,45,46], increasing the pH increases the current of the water oxidation process (Reaction (5)) and decreases the over-potential; and (ii) decreasing the pattern pitch dimensions increases the current and decreases the over-potential. After each experiment, the solution was checked by ICP, and no Ni and other metals were found. If there are impurities in the solutions, their concentrations are below the limit of detection.

The literature provides an explanation for the mechanism of the WSP [24,27,43]. Part of the mechanism regards the relationship between the Ni(OH)_2_ clusters. As a result that the clusters affect each other [27,29], the distances between them are significant and may affect reaction (5). Our findings prove, for the first time, to the best of our knowledge, that the distance between the clusters indeed affects the reaction. We have been able to demonstrate this by using DPN that enables control over the distance between the clusters.

The hydroxyl anions have two roles in this process; one in nickel oxidation (Reaction (3)) and another in oxygen formation (Reaction (5)). For Reaction (3) to be efficient, the surface area of the Ni(OH)_2_ clusters needs to be large, such that more hydroxyl anions are consumed per cluster and will react more easily and with a higher probability with Ni(OH)_2_ [31]. For Reaction (5) to be efficient, the pattern pitch dimensions needs to be ideal, so as to allow the two hydroxyls to encounter each other with a sufficiently high probability [27]. The DPN method can be used to tightly control both these features.

**Figure 4:**
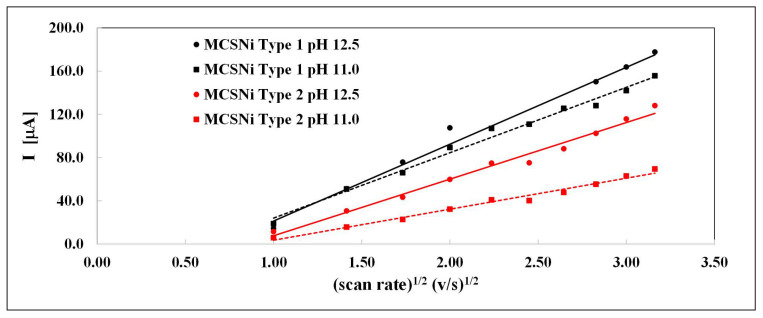
Current as a function of the square of the scan rate of the two types of MCS${}_{\mathrm{Ni}}$ electrodes (black: Type 1, red: Type 2), at two pH values (circular data points, solid line: pH 12.5, square data points, dashed line: pH 11.0, Ar atmosphere).

The linear relationship between the current of Ni(OH)_2_ oxidation (Reaction 3) and the square of the scan rate (Figure 4) indicates that the reaction is diffusion-controlled, while the diffusion coefficient depends on the pH and pattern pitch dimension. The linear curve does not pass through zero (Figure 4), indicating that the diffusion process is complex and possibly involves another, parallel reaction. This linear tendency was previously described in other processes [47]. We calculated the diffusion coefficient (D) at different pH and pattern pitch dimensions by using the Randles–Sevcik equation; the linear relationships are shown in Figure 4 and the diffusion coefficients are summarized in Table 1.

The diffusion coefficient values and their positive linear correlation with pH are similar to those reported previously [35], and indicate that the pattern pitch dimension affects the diffusion and, accordingly, the rate of the entire process; as the distance between clusters decreases, the rate of the process increases.

**Figure 5:**
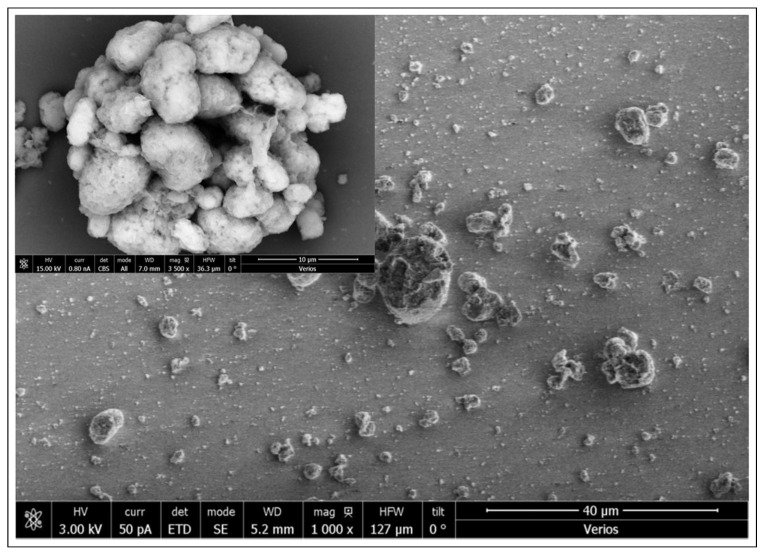
SEM image of the ITO${}_{\mathrm{Ni}}$ surface.

To compare the diffusion rates of the MCS${}_{\mathrm{Ni}}$ with those of randomly arranged Ni(OH)_2_ clusters, an ITO slice with the same surface area as that of MCS${}_{\mathrm{Ni}}$ was immersed in a saturated Ni(OH)_2_ solution for one month, until the surface was randomly coated; we refer to this electrode as ITO${}_{\mathrm{Ni}}$. The clusters that formed on the ITO${}_{\mathrm{Ni}}$ electrode were denser than those that formed on the MCS${}_{\mathrm{Ni}}$ electrode (Figure 5 inset), and with wider particle-size distribution, ranging from 100 nm to approximately 10 μm (Figure 5). The diffusion coefficients of ITO${}_{\mathrm{Ni}}$ and MSC${}_{\mathrm{Ni}}$ Type 1, Table 1, were of the same order of magnitude as the two electrodes were tested under the same conditions. However, the amounts of the catalyst used to generate the ITO${}_{\mathrm{Ni}}$ electrode were much higher than those used to create the MCS${}_{\mathrm{Ni}}$ electrode, an essential factor when the active species are expensive. This finding indicates that the MCS${}_{\mathrm{Ni}}$ electrodes are more efficient than the ITO${}_{\mathrm{Ni}}$ electrode and proves that the size of the clusters and the pattern pitch dimensions—two parameters that can be easily controlled by using DPN—dramatically affect the WSP efficiency. The clusters cannot be too close to each other (e.g., by coating the entire surface with the catalyst), since (a) the surface itself participated in some of the chemical reactions [29,46], and (b) the clusters must be in the nanoscale range, such that their surface area is sufficiently high.

## 3. Conclusions

We show that MCS${}_{\mathrm{Ni}}$ electrode can efficiently oxidize water and increase the efficiency of the WSP. The use of DPN to generate the electrode, demonstrated here for the first time, is considerably simpler than other methods [20,22,26,27]. It enables the patterning of uniform nanoclusters of OER catalysts, such that the process can be controlled by changing the solution pH, the size of the clusters, and the pattern pitch dimensions. Importantly, the MCS developed in this study also enables the highly accurate patterning of various other nano-scale materials, at extremely low quantities.

## Figures and Tables

**Table 1:** Diffusion coefficients (D) in the three electrode configurations, under two pH conditions in the electrochemistry cell *.

Electrode	Dimensions [μm]		pH	D [cm${}^{2}$/s]
	*x*-Axis	*y*-Axis		
MCS${}_{\mathrm{Ni}}$ Type 1	2.5	2.2	12.5	$1.34\times 10^{-15}$
			11.0	$1.23\times 10^{-15}$
MCS${}_{\mathrm{Ni}}$ Type 2	5.0	4.4	12.5	$7.26\times 10^{-16}$
			11.0	$2.18\times 10^{-16}$
ITO${}_{\mathrm{Ni}}$	**—**	**—**	11.0	$7.83\times 10^{-15}$

* Ionic strength =0.20 M, Ni(OH)_2_ density =0.67 gr/mL [35] electrode surface =1 cm${}^{2}$.

## Abbreviations

The following abbreviations are used in this manuscript:

WSP	Water-splitting process
OER	Oxygen-evolution reaction
HER	Hydrogen-evolution reaction
DPN	Dip-Pen Nanolithography
MCS	Meta-chemical surface
AFM	Atomic force microscopy
SEM	Scanning electron microscopy
EDS	Energy-dispersive X-ray spectroscopy
XRD	X-ray diffraction
ITO	Indium tin oxide
LSV	Linear sweep voltammetry
